# Supplementary material for: Promoting Evidence Based Nutrition Education Across the World in a Competitive Space: Delivering a Massive Open Online Course
Source: Nutrients. 2020 Jan 28;12(2):344. doi: 10.3390/nu12020344 (PMC7071325; doi:10.3390/nu12020344)
Supplement: Supplementary file 1 [file nutrients-12-00344-s001.pdf]

Table S1. Course Outline.

Week	Theme	Topic/Content	Delivery Mode
1: Food as Medicine	Introduction	Welcome to the Course<br>Course Philosophy	Videos and written articles
	History of Food as Medicine	Food as Medicine in History	Videos and written articles
		Importance of evidence	Article
	Food in Health and Disease	The role food can play in prevention and treatment	Videos and written articles
		How many different foods you consume everyday?	Activity
	What's in food that makes it special?	Food matrix, macro and micronutrients, phytochemicals, using supplements	Videos and written articles
		Superfoods Quiz	Quiz
		Superfoods: Myth or Real	Activity and discussion
	Foods and Inflammation	Acute and chronic inflammation<br>Foods and inflammation	Videos and written articles
	Weekly feedback		Video
2: A Body System's Approach to Food as Medicine	Food and the Gut	Prebiotics, probiotics, fibre, FODMAPS	Videos and written articles
	Food and the Brain	Food and appetite<br>Food addiction	Videos, written articles and surveys
	Food and our Genome	What are genes and why are they important for our health?<br>Food, nutrition and our genome	Videos, written articles, quiz and discussion points
	Food, Fertility and Pregnancy	Nutrition and fertility, pregnancy and total energy intake	Videos, written articles, quiz, survey and discussion points
	Food and Weight	Classifying weight and BMI, weight regain, complexity of achieving healthy weight, diets	Videos and written articles
	Weekly feedback		Video
3: Interpreting the Science of Food as Medicine	Nutrition Complexities and Controversies	Factors influencing what we eat, evolution of nutrition science, how misinformation can end up as fact, popular diets	Videos, written articles, discussions and activity
	Making Choices: Foods and Diet	The challenges with portion sizes, local and international dietary guidelines	Videos, written articles, quiz and discussions
	Who Can you Trust	Nutrition Information- what to look out for?	Video
		Evidence and nutrition advice	Activity
	Where is the future heading?	Functional foods, personalized medicine	Videos, written articles and discussions
	Weekly Feedback		Video
	End of course	End of course discussion	Discussion
		Supporting information	Article

Table S2. Qualitative Research Review Guidelines (RATS) checklist.

Qualitative Research Review Guidelines – RATS [1]		
Ask this of the manuscript	This should be included in the manuscript	Where item has been addressed
R – Relevance of study design		
Is research question interesting?	Research question explicitly stated	P2 (end of introduction)
Is research question relevant to clinical practice, public health, or policy?	Research question justified and linked to the existing knowledge base (empirical research, theory, policy)	P1-2
A – Appropriateness of qualitative method		
Is qualitative methodology the best approach for the study<br>aims?<br>*Interviews*: experience, perceptions, behavior, practice, process<br>*Focus groups*: group dynamics, convenience, non-sensitive topics<br>*Ethnography*: culture, organizational behavior, interaction<br>*Textual analysis*: documents, art, representations, conversations	Study design described and justified e.g., why was a particular method (i.e., interviews) chosen?	P2-3
T – transparency of procedures		
		P3

Sampling<br><br>Are the participants selected the most appropriate to provide access to the type of knowledge sought by the study?<br><br>Is the sampling strategy appropriate?	Criteria for selecting the study sample justified and explained<br><br>*theoretical*: based on pre conceived or emergent theory<br><br>*purposive*: diversity of opinion<br><br>*volunteer*: feasibility, hard-to-reach groups	
Recruitment<br><br>Was recruitment conducted using appropriate methods?<br><br>Is the sampling strategy appropriate?<br><br>Could there be selection bias?	Details of how recruitment was conducted and by whom<br><br>Details of who chose not to participate and why	P3<br><br>P3
Data collection<br><br>Was collection of data systematic and comprehensive?<br><br>Are characteristics of study group and setting clear?<br><br>Why and when was data collection stopped, and is this reasonable?	Method (s) outlined and examples given (e.g., interview questions)<br><br>Study group and setting clearly described<br><br>End of data collection justified and described	P3<br><br>P3<br><br>Page 3
Role of researchers<br><br>Is the researcher(s) appropriate?	Do the researchers occupy dual roles (clinician and researcher)?	Page 3

How might they bias (good and bad) the conduct of the study and results?	Are the ethics of this discussed? Do the researcher(s) critically examine their own influence on the formulation of the research question, data collection, and interpretation?	Page3
Ethics<br><br>Was informed consent sought and granted?<br><br>Were participants'' anonymity and confidentiality ensured?<br><br>Was approval from an appropriate ethics committee received?	Informed consent process explicitly and dearly detailed<br><br>Anonymity and confidentiality discussed<br><br>Ethics approval cited	Page 3<br><br>Page 8<br><br>Page 8
S - Soundness of interpretive approach<br><br>*Analysis*<br><br>Is the type of analysis appropriate for the type of study?<br><br>*Thematic*: exploratory, descriptive, hypothesis generating<br><br>*Framework:* e.g. policy<br><br>*Constant comparison/grounded theory:* theory generating, analytical	Analytic approach described in depth and justified<br><br>Indicators of quality: Description of how themes were developed from the data (inductive or deductive)<br><br>Evidence of alternative explanations being sought<br><br>Analysis and presentation of negative or deviant cases<br><br>Description of the basis on which quotes were chosen	Page 3<br><br>Page 3<br><br>Page 3

Are the interpretations dearly presented and adequately supported by the evidence?<br><br>Are quotes used and are these appropriate and effective?<br><br>Was trustworthiness/reliability of the data and interpretations checked?	Semi-quantification when appropriate<br><br>Illumination of context and/or meaning, richly detailed<br><br>Method of reliability check described and justified<br><br>e.g., was an audit trail, triangulation, or<br><br>member checking employed?<br><br>Did an independent analyst review data and contest themes? How were disagreements resolved?	Pages 4-5<br><br>Pages 4-5<br><br>Page 3
Discussion and presentation<br><br>Are findings sufficiently grounded in a theoretical or conceptual framework?<br><br>Is adequate account taken of previous knowledge and how the findings add?<br><br>Are the limitations thoughtfully considered?	Findings presented with reference to existing<br><br>theoretical and empirical literature, and how they contribute<br><br>Strengths and limitations explicitly described and discussed	Pages 7-9<br><br>Pages 7-9<br><br>Page 9

Is the manuscript well written and accessible?	Evidence following guidelines (format, word count)	Title page
	Detail of methods or additional quotes, contained in appendix	No
	Written for a health sciences audience	Yes
Are red flags present?<br><br>These are common features of ill-conceived or poorly executed qualitative studies, are a cause for concern, and must be viewed critically<br><br>They might be fatal flaws, or they may result from lack of detail or clarity	Grounded theory; not a simple content analysis but a complex, sociological, theory generating approach<br><br>*Jargon*: descriptions that are trite or jargon filled should be viewed skeptically<br><br>*Over interpretation*: interpretation must be grounded in "accounts" and semi-quantified if possible or appropriate<br><br>*Seems anecdotal, self-evident*: may be a superficial analysis, not rooted in conceptual framework or linked to previous knowledge, and lacking depth<br><br>*Consent process thinly discussed*: may not have met ethics requirements	-

	Doctor-researcher: consider the ethical implications for patients and the bias in data collection and interpretation

[1] The RATS guidelines modified for BioMed Central are copyright Jocalyn Clark, BMJ. They can be found in Clark JP: How to peer review a qualitative manuscript. In *Peer Review Health Sciences*. Second edition. Edited by Godlee F, Jefferson T. London: BMJ Books; 2003:219-235.
